# 1-[(2*S*)-1-Chloro-3-phenyl­propan-2-yl]-2,4,5-triphenyl-1*H*-imidazole

**DOI:** 10.1107/S1600536812009609

**Published:** 2012-03-24

**Authors:** Yongmei Xiao, Liangru Yang, Kun He, Jinwei Yuan, Pu Mao

**Affiliations:** a School of Chemistry and Chemical Engineering, Henan University of Technology, Zhengzhou 450001, People’s Republic of China

## Abstract

In the title compound, C_30_H_25_ClN_2_, the chiral center maintains the *S* configuration of the stating l-phenyl­alaninol. The two phenyl groups closest to the substituted N atom adopt an almost perpendicular orientation relative to the central imidazole ring, with dihedral angles of 88.9 (4) and 84.7 (3)°. The third phenyl group is nearly coplanar with it, making a dihedral angle of 11.0 (5)°.

## Related literature

For the synthesis and applications of chiral ionic liquids, see: Ding *et al.* (2005 ▶); Bwambok *et al.* (2008 ▶); Mao *et al.* (2010 ▶).
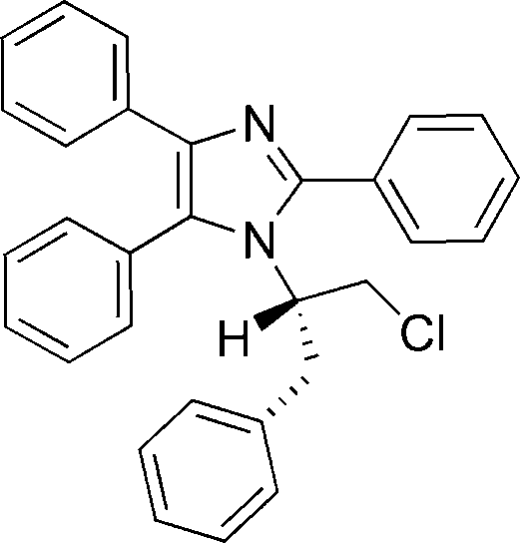


## Experimental

### 

#### Crystal data

C_30_H_25_ClN_2_
*M* *_r_* = 448.97
Orthorhombic, 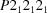

*a* = 9.6123 (4) Å
*b* = 9.9437 (3) Å
*c* = 24.9677 (7) Å
*V* = 2386.47 (14) Å^3^
*Z* = 4
Cu *K*α radiation
μ = 1.56 mm^−1^
*T* = 291 K
0.21 × 0.20 × 0.06 mm

#### Data collection

Agilent Xcalibur Eos Gemini diffractometer
Absorption correction: multi-scan (*CrysAlis PRO*; Agilent, 2011 ▶) *T*_min_ = 0.657, *T*_max_ = 1.000
9379 measured reflections
4256 independent reflections
3235 reflections with *I* > 2σ(*I*)
*R*_int_ = 0.044

#### Refinement

*R*[*F* ^2^ > 2σ(*F* ^2^)] = 0.045
*wR*(*F* ^2^) = 0.098
*S* = 1.02
4256 reflections
298 parameters
H-atom parameters constrained
Δρ_max_ = 0.14 e Å^−3^
Δρ_min_ = −0.15 e Å^−3^
Absolute structure: Flack (1983 ▶), 1816 Friedel pairs
Flack parameter: 0.01 (2)

### 

Data collection: *CrysAlis PRO* (Agilent, 2011 ▶); cell refinement: *CrysAlis PRO*; data reduction: *CrysAlis PRO*; program(s) used to solve structure: *SHELXS97* (Sheldrick, 2008 ▶); program(s) used to refine structure: *SHELXL97* (Sheldrick, 2008 ▶); molecular graphics: *OLEX2* (Dolomanov *et al.*, 2009 ▶); software used to prepare material for publication: *OLEX2*.

## Supplementary Material

Crystal structure: contains datablock(s) I, global. DOI: 10.1107/S1600536812009609/ld2044sup1.cif

Structure factors: contains datablock(s) I. DOI: 10.1107/S1600536812009609/ld2044Isup2.hkl

Supplementary material file. DOI: 10.1107/S1600536812009609/ld2044Isup3.cml

Additional supplementary materials:  crystallographic information; 3D view; checkCIF report

## Figures and Tables

**Fig. 1.**
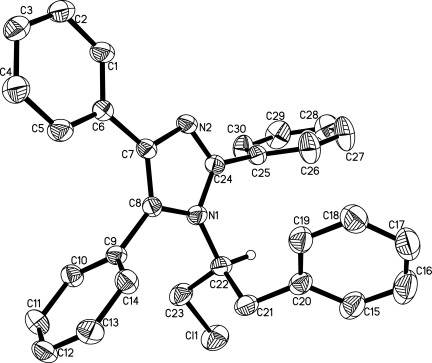
The molecular structure of the title compound showing 30% probability displacement ellipsoids. Hydrogen atoms are omitted for clarity.

## supplementary crystallographic information

### Comment

Current interest in the stereoselective synthesis and catalysis, chiral recognization and separation in ionic liquids has motivated the synthesis of novel chiral ionic liquids. (Ding & Armstrong, 2005; Bwambok *et al.*, 2008). Our group is interested in the preparation and application of chiral imidazolium derivatives from natural precursors (Mao *et al.*, 2010). During the study, we observed that condensation of *l*-phenylalaninol, dibenzoyl, arylaldehyde and ammonium acetate afforded multi-aryl substituted imidazole alcohol derivatives carrying a chiral functionality. The following reaction with SOCl_2_ produced the title compound smoothly.

The molecular structure of the title compound is shown in Figure 1. As it is expected, the imidazole core (N1, C8, C7, N2, C24) is essentially planar. featuring an average deviation of less than 0.6 (3) °. The dihedral angles formed by the three aryl substituents and the central imidazole ring are 88.9 (4) (N2—C24—C25—C26), 11.0 (5) (C5—C6—C7—C8) and 95.3 (3) ° (C7—C8—C9—C14).

Due to the presence of muti aryl groups on the imidazole ring, the basicity of the N2 of the imidazole is reduced and its quaternization by the produced chloro- substituted derivative is suppressed successfully.

### Experimental

SOCl_2_ (40 ml) was added slowly at room temperature into a three-neck flask containing 2-(4,5-Diphenyl-2-*p*-tolyl-imidazol-1-yl)-3-phenyl-propan-1-ol (4.45 g, 0.01 mol) and Na_2_CO_3_ (1.06 g, 0.01 mol). The solids were slowly dissolved upon addition of SOCl_2_. After complete addition, the mixture was kept at 50 °C for 5 h and then at 70 °C for 2 h. The excessive SOCl_2_ was removed and the residue was washed with H_2_O and filtered to afford the crude product. Crystallization from EtOH afforded colorless crystals of the title compound.

### Refinement

A suitable crystal was selected and mounted on a Xcalibur, Eos, Gemini diffractometer.The crystal was kept at 291.15 K during data collection. Using Olex2 (Dolomanov *et al.*, 2009), the structure was solved with the *SHELXS* (Sheldrick, 2008) structure solution program using Direct Methods and refined with the *SHELXL* (Sheldrick, 2008) refinement package using Least Squares minimization.

### Crystal data

**Table:**

C_30_H_25_ClN_2_	*D*_x_ = 1.250 Mg m^−^^3^
*M**_r_* = 448.97	Cu *K*α radiation, λ = 1.5418 Å
Orthorhombic, *P*2_1_2_1_2_1_	Cell parameters from 2343 reflections
*a* = 9.6123 (4) Å	θ = 3.5–66.9°
*b* = 9.9437 (3) Å	µ = 1.56 mm^−^^1^
*c* = 24.9677 (7) Å	*T* = 291 K
*V* = 2386.47 (14) Å^3^	Prismatic, colorless
*Z* = 4	0.21 × 0.20 × 0.06 mm
*F*(000) = 944	

### Data collection

**Table:**

Agilent Xcalibur Eos Gemini diffractometer	4256 independent reflections
Radiation source: Enhance (Cu) X-ray Source	3235 reflections with *I* > 2σ(*I*)
Graphite monochromator	*R*_int_ = 0.044
Detector resolution: 16.2312 pixels mm^-1^	θ_max_ = 67.0°, θ_min_ = 3.5°
ω scans	*h* = −8→11
Absorption correction: multi-scan (*CrysAlis PRO*; Agilent, 2011)	*k* = −11→11
*T*_min_ = 0.657, *T*_max_ = 1.000	*l* = −29→29
9379 measured reflections	

### Refinement

**Table:**

Refinement on *F*^2^	Secondary atom site location: difference Fourier map
Least-squares matrix: full	Hydrogen site location: inferred from neighbouring sites
*R*[*F*^2^ > 2σ(*F*^2^)] = 0.045	H-atom parameters constrained
*wR*(*F*^2^) = 0.098	*w* = 1/[σ^2^(*F*_o_^2^) + (0.0291*P*)^2^] where *P* = (*F*_o_^2^ + 2*F*_c_^2^)/3
*S* = 1.02	(Δ/σ)_max_ = 0.001
4256 reflections	Δρ_max_ = 0.14 e Å^−^^3^
298 parameters	Δρ_min_ = −0.15 e Å^−^^3^
0 restraints	Absolute structure: Flack (1983), 1816 Friedel pairs
Primary atom site location: structure-invariant direct methods	Flack parameter: 0.01 (2)

### Special details

**Table:**

Geometry. All e.s.d.'s (except the e.s.d. in the dihedral angle between two l.s. planes) are estimated using the full covariance matrix. The cell e.s.d.'s are taken into account individually in the estimation of e.s.d.'s in distances, angles and torsion angles; correlations between e.s.d.'s in cell parameters are only used when they are defined by crystal symmetry. An approximate (isotropic) treatment of cell e.s.d.'s is used for estimating e.s.d.'s involving l.s. planes.
Refinement. Refinement of *F*^2^ against ALL reflections. The weighted *R*-factor *wR* and goodness of fit *S* are based on *F*^2^, conventional *R*-factors *R* are based on *F*, with *F* set to zero for negative *F*^2^. The threshold expression of *F*^2^ > σ(*F*^2^) is used only for calculating *R*-factors(gt) *etc*. and is not relevant to the choice of reflections for refinement. *R*-factors based on *F*^2^ are statistically about twice as large as those based on *F*, and *R*- factors based on ALL data will be even larger.

### Fractional atomic coordinates and isotropic or equivalent isotropic displacement parameters (Å^2^)

**Table:**

	*x*	*y*	*z*	*U*_iso_*/*U*_eq_	
Cl1	0.02166 (10)	0.49291 (10)	0.54575 (3)	0.0805 (3)	
N1	0.2271 (2)	0.64694 (19)	0.67166 (8)	0.0430 (5)	
N2	0.2501 (3)	0.80764 (18)	0.73267 (8)	0.0433 (5)	
C1	0.2662 (3)	0.7993 (3)	0.84491 (10)	0.0525 (7)	
H1	0.2457	0.8782	0.8266	0.063*	
C2	0.2834 (4)	0.8033 (3)	0.90011 (11)	0.0619 (8)	
H2	0.2755	0.8844	0.9184	0.074*	
C3	0.3121 (4)	0.6867 (3)	0.92756 (10)	0.0617 (8)	
H3	0.3223	0.6884	0.9646	0.074*	
C4	0.3255 (4)	0.5675 (3)	0.90000 (11)	0.0626 (8)	
H4	0.3461	0.4887	0.9185	0.075*	
C5	0.3085 (3)	0.5641 (3)	0.84478 (11)	0.0545 (8)	
H5	0.3171	0.4830	0.8266	0.065*	
C6	0.2790 (3)	0.6806 (2)	0.81663 (9)	0.0415 (5)	
C7	0.2604 (3)	0.6838 (2)	0.75749 (9)	0.0395 (5)	
C8	0.2469 (3)	0.5829 (2)	0.72076 (9)	0.0382 (5)	
C9	0.2431 (3)	0.4337 (2)	0.72724 (9)	0.0394 (6)	
C10	0.1175 (3)	0.3689 (3)	0.73505 (11)	0.0477 (6)	
H10	0.0357	0.4187	0.7364	0.057*	
C11	0.1120 (3)	0.2302 (3)	0.74093 (12)	0.0564 (8)	
H11	0.0269	0.1877	0.7461	0.068*	
C12	0.2317 (4)	0.1558 (2)	0.73909 (12)	0.0574 (8)	
H12	0.2280	0.0628	0.7425	0.069*	
C13	0.3575 (3)	0.2192 (3)	0.73211 (12)	0.0556 (7)	
H13	0.4388	0.1686	0.7312	0.067*	
C14	0.3644 (3)	0.3580 (3)	0.72642 (11)	0.0494 (7)	
H14	0.4501	0.4000	0.7221	0.059*	
C15	0.5054 (4)	0.6007 (3)	0.53636 (12)	0.0726 (10)	
H15	0.4501	0.5715	0.5081	0.087*	
C16	0.6292 (5)	0.6662 (4)	0.52570 (16)	0.0907 (13)	
H16	0.6567	0.6799	0.4904	0.109*	
C17	0.7110 (4)	0.7106 (3)	0.56644 (17)	0.0845 (12)	
H17	0.7943	0.7544	0.5590	0.101*	
C18	0.6708 (4)	0.6909 (3)	0.61861 (16)	0.0719 (9)	
H18	0.7260	0.7218	0.6466	0.086*	
C19	0.5479 (3)	0.6250 (3)	0.62902 (12)	0.0611 (8)	
H19	0.5210	0.6114	0.6644	0.073*	
C20	0.4629 (3)	0.5783 (3)	0.58816 (10)	0.0521 (7)	
C21	0.3307 (3)	0.5022 (3)	0.59933 (10)	0.0539 (7)	
H21A	0.3494	0.4355	0.6267	0.065*	
H21B	0.3036	0.4546	0.5671	0.065*	
C22	0.2082 (3)	0.5894 (3)	0.61769 (10)	0.0482 (7)	
H22	0.2023	0.6653	0.5927	0.058*	
C23	0.0692 (3)	0.5147 (3)	0.61442 (10)	0.0612 (8)	
H23A	−0.0023	0.5657	0.6329	0.073*	
H23B	0.0773	0.4277	0.6317	0.073*	
C24	0.2292 (3)	0.7824 (2)	0.68147 (10)	0.0414 (5)	
C25	0.2084 (3)	0.8899 (2)	0.64130 (10)	0.0449 (6)	
C26	0.3191 (4)	0.9466 (3)	0.61519 (14)	0.0750 (10)	
H26	0.4076	0.9104	0.6197	0.090*	
C27	0.3010 (5)	1.0571 (4)	0.58219 (16)	0.0921 (13)	
H27	0.3770	1.0946	0.5646	0.111*	
C28	0.1709 (4)	1.1112 (3)	0.57538 (13)	0.0762 (11)	
H28	0.1585	1.1856	0.5533	0.091*	
C29	0.0601 (4)	1.0555 (4)	0.60104 (14)	0.0781 (11)	
H29	−0.0283	1.0919	0.5965	0.094*	
C30	0.0785 (3)	0.9450 (3)	0.63390 (12)	0.0640 (8)	
H30	0.0021	0.9074	0.6512	0.077*	

### Atomic displacement parameters (Å^2^)

**Table:**

	*U*^11^	*U*^22^	*U*^33^	*U*^12^	*U*^13^	*U*^23^
Cl1	0.0822 (6)	0.1023 (6)	0.0571 (3)	−0.0076 (6)	−0.0157 (4)	−0.0137 (5)
N1	0.0504 (13)	0.0381 (10)	0.0404 (10)	0.0030 (10)	0.0030 (10)	−0.0012 (9)
N2	0.0537 (13)	0.0320 (9)	0.0441 (10)	−0.0012 (11)	−0.0006 (11)	−0.0012 (9)
C1	0.0667 (19)	0.0384 (12)	0.0524 (13)	−0.0001 (16)	−0.0016 (15)	−0.0048 (12)
C2	0.081 (2)	0.0531 (15)	0.0520 (14)	−0.0010 (18)	−0.0013 (16)	−0.0120 (13)
C3	0.077 (2)	0.0647 (18)	0.0431 (13)	−0.0035 (18)	−0.0066 (14)	−0.0035 (14)
C4	0.082 (2)	0.0540 (15)	0.0517 (14)	0.0087 (17)	−0.0078 (15)	0.0070 (14)
C5	0.074 (2)	0.0402 (13)	0.0491 (13)	0.0059 (15)	−0.0017 (14)	−0.0030 (12)
C6	0.0432 (13)	0.0391 (12)	0.0422 (11)	−0.0024 (12)	−0.0001 (11)	0.0001 (10)
C7	0.0406 (14)	0.0329 (10)	0.0449 (12)	0.0006 (12)	−0.0001 (12)	0.0004 (10)
C8	0.0367 (13)	0.0345 (11)	0.0435 (12)	0.0020 (11)	0.0045 (12)	0.0006 (10)
C9	0.0466 (15)	0.0331 (10)	0.0384 (11)	0.0026 (12)	0.0011 (12)	−0.0022 (9)
C10	0.0459 (15)	0.0394 (14)	0.0579 (14)	0.0057 (13)	0.0043 (14)	0.0019 (13)
C11	0.0532 (17)	0.0477 (16)	0.0683 (18)	−0.0100 (14)	−0.0030 (16)	0.0052 (15)
C12	0.079 (2)	0.0311 (11)	0.0618 (15)	−0.0025 (15)	−0.0026 (18)	0.0010 (12)
C13	0.0596 (18)	0.0421 (16)	0.0650 (17)	0.0192 (15)	0.0023 (16)	0.0022 (14)
C14	0.0441 (15)	0.0445 (15)	0.0596 (15)	0.0011 (13)	0.0062 (14)	0.0021 (13)
C15	0.092 (3)	0.073 (2)	0.0528 (16)	0.011 (2)	0.0135 (18)	0.0050 (15)
C16	0.108 (3)	0.092 (3)	0.072 (2)	0.008 (3)	0.034 (2)	0.023 (2)
C17	0.079 (3)	0.0602 (19)	0.114 (3)	0.001 (2)	0.036 (3)	0.014 (2)
C18	0.070 (2)	0.0579 (17)	0.087 (2)	−0.0023 (18)	0.0098 (19)	0.0003 (18)
C19	0.070 (2)	0.0577 (16)	0.0555 (15)	0.0019 (17)	0.0098 (16)	0.0060 (15)
C20	0.0629 (19)	0.0471 (15)	0.0464 (13)	0.0125 (15)	0.0084 (14)	0.0004 (12)
C21	0.0678 (19)	0.0488 (14)	0.0451 (12)	0.0048 (17)	0.0049 (13)	−0.0073 (13)
C22	0.0606 (18)	0.0424 (13)	0.0417 (12)	−0.0012 (14)	0.0010 (13)	−0.0041 (11)
C23	0.0660 (19)	0.076 (2)	0.0416 (12)	−0.0059 (19)	−0.0029 (13)	−0.0096 (15)
C24	0.0453 (14)	0.0342 (11)	0.0448 (12)	0.0020 (13)	0.0022 (12)	0.0003 (10)
C25	0.0547 (16)	0.0388 (12)	0.0412 (12)	−0.0009 (13)	0.0015 (12)	0.0020 (11)
C26	0.063 (2)	0.073 (2)	0.089 (2)	0.0052 (18)	0.0099 (19)	0.0319 (19)
C27	0.090 (3)	0.088 (3)	0.098 (3)	−0.010 (2)	0.015 (2)	0.048 (2)
C28	0.107 (3)	0.0606 (19)	0.0615 (18)	0.012 (2)	−0.003 (2)	0.0232 (16)
C29	0.084 (3)	0.079 (2)	0.0713 (19)	0.030 (2)	−0.004 (2)	0.0219 (18)
C30	0.0601 (19)	0.0696 (19)	0.0623 (16)	0.0061 (16)	0.0063 (16)	0.0164 (16)

### Geometric parameters (Å, º)

**Table:**

Cl1—C23	1.788 (2)	C15—H15	0.9300
N1—C8	1.395 (3)	C15—C16	1.383 (6)
N1—C22	1.475 (3)	C15—C20	1.374 (4)
N1—C24	1.370 (3)	C16—H16	0.9300
N2—C7	1.382 (3)	C16—C17	1.360 (6)
N2—C24	1.318 (3)	C17—H17	0.9300
C1—H1	0.9300	C17—C18	1.373 (5)
C1—C2	1.389 (4)	C18—H18	0.9300
C1—C6	1.380 (3)	C18—C19	1.376 (5)
C2—H2	0.9300	C19—H19	0.9300
C2—C3	1.374 (4)	C19—C20	1.387 (4)
C3—H3	0.9300	C20—C21	1.506 (4)
C3—C4	1.377 (4)	C21—H21A	0.9700
C4—H4	0.9300	C21—H21B	0.9700
C4—C5	1.389 (4)	C21—C22	1.532 (4)
C5—H5	0.9300	C22—H22	0.9800
C5—C6	1.384 (3)	C22—C23	1.531 (4)
C6—C7	1.488 (3)	C23—H23A	0.9700
C7—C8	1.366 (3)	C23—H23B	0.9700
C8—C9	1.492 (3)	C24—C25	1.479 (3)
C9—C10	1.383 (4)	C25—C26	1.370 (4)
C9—C14	1.388 (4)	C25—C30	1.376 (4)
C10—H10	0.9300	C26—H26	0.9300
C10—C11	1.388 (4)	C26—C27	1.384 (4)
C11—H11	0.9300	C27—H27	0.9300
C11—C12	1.369 (4)	C27—C28	1.372 (5)
C12—H12	0.9300	C28—H28	0.9300
C12—C13	1.374 (5)	C28—C29	1.361 (5)
C13—H13	0.9300	C29—H29	0.9300
C13—C14	1.389 (4)	C29—C30	1.383 (4)
C14—H14	0.9300	C30—H30	0.9300
			
C8—N1—C22	130.0 (2)	C16—C17—H17	120.0
C24—N1—C8	106.9 (2)	C16—C17—C18	120.0 (4)
C24—N1—C22	123.1 (2)	C18—C17—H17	120.0
C24—N2—C7	106.05 (19)	C17—C18—H18	120.3
C2—C1—H1	119.3	C17—C18—C19	119.3 (4)
C6—C1—H1	119.3	C19—C18—H18	120.3
C6—C1—C2	121.4 (3)	C18—C19—H19	119.1
C1—C2—H2	120.2	C18—C19—C20	121.7 (3)
C3—C2—C1	119.6 (3)	C20—C19—H19	119.1
C3—C2—H2	120.2	C15—C20—C19	117.6 (3)
C2—C3—H3	120.1	C15—C20—C21	120.4 (3)
C2—C3—C4	119.7 (3)	C19—C20—C21	121.9 (2)
C4—C3—H3	120.1	C20—C21—H21A	108.6
C3—C4—H4	119.8	C20—C21—H21B	108.6
C3—C4—C5	120.4 (3)	C20—C21—C22	114.8 (2)
C5—C4—H4	119.8	H21A—C21—H21B	107.5
C4—C5—H5	119.7	C22—C21—H21A	108.6
C6—C5—C4	120.6 (3)	C22—C21—H21B	108.6
C6—C5—H5	119.7	N1—C22—C21	113.4 (2)
C1—C6—C5	118.3 (2)	N1—C22—H22	106.8
C1—C6—C7	118.6 (2)	N1—C22—C23	110.2 (2)
C5—C6—C7	123.1 (2)	C21—C22—H22	106.8
N2—C7—C6	118.23 (19)	C23—C22—C21	112.3 (2)
C8—C7—N2	110.3 (2)	C23—C22—H22	106.8
C8—C7—C6	131.4 (2)	Cl1—C23—H23A	109.8
N1—C8—C9	123.1 (2)	Cl1—C23—H23B	109.8
C7—C8—N1	105.5 (2)	C22—C23—Cl1	109.44 (19)
C7—C8—C9	131.3 (2)	C22—C23—H23A	109.8
C10—C9—C8	120.0 (3)	C22—C23—H23B	109.8
C10—C9—C14	118.8 (2)	H23A—C23—H23B	108.2
C14—C9—C8	121.2 (3)	N1—C24—C25	126.0 (2)
C9—C10—H10	119.6	N2—C24—N1	111.3 (2)
C9—C10—C11	120.8 (3)	N2—C24—C25	122.8 (2)
C11—C10—H10	119.6	C26—C25—C24	121.0 (3)
C10—C11—H11	120.0	C26—C25—C30	118.5 (3)
C12—C11—C10	120.1 (3)	C30—C25—C24	120.1 (3)
C12—C11—H11	120.0	C25—C26—H26	119.6
C11—C12—H12	120.1	C25—C26—C27	120.8 (3)
C11—C12—C13	119.8 (2)	C27—C26—H26	119.6
C13—C12—H12	120.1	C26—C27—H27	120.0
C12—C13—H13	119.6	C28—C27—C26	120.0 (4)
C12—C13—C14	120.7 (3)	C28—C27—H27	120.0
C14—C13—H13	119.6	C27—C28—H28	120.2
C9—C14—C13	119.8 (3)	C29—C28—C27	119.7 (3)
C9—C14—H14	120.1	C29—C28—H28	120.2
C13—C14—H14	120.1	C28—C29—H29	119.9
C16—C15—H15	119.6	C28—C29—C30	120.2 (3)
C20—C15—H15	119.6	C30—C29—H29	119.9
C20—C15—C16	120.9 (4)	C25—C30—C29	120.8 (3)
C15—C16—H16	119.8	C25—C30—H30	119.6
C17—C16—C15	120.5 (3)	C29—C30—H30	119.6
C17—C16—H16	119.8		
			
N1—C8—C9—C10	−85.7 (3)	C10—C11—C12—C13	0.9 (5)
N1—C8—C9—C14	95.3 (3)	C11—C12—C13—C14	−0.6 (5)
N1—C22—C23—Cl1	161.61 (19)	C12—C13—C14—C9	−0.6 (4)
N1—C24—C25—C26	−92.4 (4)	C14—C9—C10—C11	−1.2 (4)
N1—C24—C25—C30	94.9 (4)	C15—C16—C17—C18	−0.2 (6)
N2—C7—C8—N1	−0.2 (3)	C15—C20—C21—C22	−105.2 (3)
N2—C7—C8—C9	−176.5 (3)	C16—C15—C20—C19	0.7 (5)
N2—C24—C25—C26	88.9 (4)	C16—C15—C20—C21	−177.6 (3)
N2—C24—C25—C30	−83.8 (4)	C16—C17—C18—C19	0.6 (5)
C1—C2—C3—C4	1.0 (5)	C17—C18—C19—C20	−0.3 (5)
C1—C6—C7—N2	8.6 (4)	C18—C19—C20—C15	−0.3 (5)
C1—C6—C7—C8	−169.1 (3)	C18—C19—C20—C21	178.0 (3)
C2—C1—C6—C5	0.5 (5)	C19—C20—C21—C22	76.5 (3)
C2—C1—C6—C7	−179.5 (3)	C20—C15—C16—C17	−0.5 (6)
C2—C3—C4—C5	−0.8 (6)	C20—C21—C22—N1	−69.1 (3)
C3—C4—C5—C6	0.5 (5)	C20—C21—C22—C23	165.1 (2)
C4—C5—C6—C1	−0.3 (5)	C21—C22—C23—Cl1	−70.9 (3)
C4—C5—C6—C7	179.6 (3)	C22—N1—C8—C7	179.2 (3)
C5—C6—C7—N2	−171.3 (3)	C22—N1—C8—C9	−4.1 (5)
C5—C6—C7—C8	11.0 (5)	C22—N1—C24—N2	−178.8 (2)
C6—C1—C2—C3	−0.8 (5)	C22—N1—C24—C25	2.4 (5)
C6—C7—C8—N1	177.7 (3)	C24—N1—C8—C7	−0.3 (3)
C6—C7—C8—C9	1.3 (5)	C24—N1—C8—C9	176.5 (3)
C7—N2—C24—N1	−0.7 (3)	C24—N1—C22—C21	119.7 (3)
C7—N2—C24—C25	178.1 (3)	C24—N1—C22—C23	−113.4 (3)
C7—C8—C9—C10	90.2 (4)	C24—N2—C7—C6	−177.7 (2)
C7—C8—C9—C14	−88.8 (4)	C24—N2—C7—C8	0.5 (3)
C8—N1—C22—C21	−59.6 (4)	C24—C25—C26—C27	−172.7 (3)
C8—N1—C22—C23	67.3 (4)	C24—C25—C30—C29	172.6 (3)
C8—N1—C24—N2	0.6 (3)	C25—C26—C27—C28	0.1 (6)
C8—N1—C24—C25	−178.2 (3)	C26—C25—C30—C29	−0.3 (5)
C8—C9—C10—C11	179.7 (3)	C26—C27—C28—C29	−0.2 (6)
C8—C9—C14—C13	−179.5 (2)	C27—C28—C29—C30	0.1 (6)
C9—C10—C11—C12	0.0 (5)	C28—C29—C30—C25	0.2 (5)
C10—C9—C14—C13	1.5 (4)	C30—C25—C26—C27	0.1 (5)
